# Epidermal Growth Factor Modulates Fetal Thymocyte Growth and Differentiation

**DOI:** 10.1155/1998/46801

**Published:** 1998

**Authors:** Claudia S. Freitas, Sergio R. Dalmau, Karla Kovary, Wilson Savino

**Affiliations:** 1 Department of Immunology Basic Research Center National Cancer Institute of Rio de Janeiro Brazil; 2 Department of Biochemistry Institute of Biology Rio de Janeiro State University Brazil; 3 Laboratory on Thymus Research Oswaldo Cruz Foundation Rio de Janeiro Brazil

**Keywords:** Epidermal growth factor, fetal thymus organ culture, T-cell ontogeny

## Abstract

In the present study, we used the fetal organ culture (FTOC) technique in order to study a putative effect of epidermal growth factor (EGF) on the thymus ontogeny. Functional EGF receptors and more recently the EGF molecule itself, respectively, on the membrane of epithelial components of thymic stroma and on a few thymocytes in adult thymus, had been reported in the literature. We could observe a dose-dependent decrease in cellularity and a progressive retention of thymocytes in the double-negative (CD4^-^/CD8^-^) stage of differentiation when exogenous EGF was added. Epidermal growth factor interfered with both fetal stroma growth and thymocyte development at a precise moment, that is, in the passage from double-negative to the double-positive (CD4^+^/CD8^+^) stage. After a 7-day FTOC in the presence of EGF, most cells recovered were Thy-1.2^+^, c-kit^+^, TSA1^-/int^, CD3^-^, and one of CD44^high^/CD25^int^, CD44^-^/CD25^int^, or CD44^-^/CD25^-^. Some developed into *γδ*TCR^+^ cells with a mature (CD3^+^) phenotype, but not into *αβ*TCR^+^ thymocytes. It seems that EGF addition makes the cultures "nonpermissible" for *αβ*TCR^+^ thymocyte generation. We report here the presence of a high Mr "EGF-like" molecule on the membrane of fetal thymocytes, which role in the observed effects is under investigation. Further biochemical characterization of this molecule is still required, because its presence was only evidenced on the basis of its antigenicity.


*Developmental Immunology*, 1998, Vol. 5, pp. 169–182
Reprints available directly from the publisher
Photocopying permitted by license only

© 1998 OPA (Overseas Publishers Association)
Amsterdam B.V. Published under license
under the Harwood Academic Publishers imprint,
part of the Gordon and Breach Publishing Group
Printed in Malaysia

### Epidermal Growth Factor Modulates Fetal Thymocyte Growth and Differentiation

CLAUDIA S. FREITAS[a*], SERGIO R. DALMAU[a,b], KARLA KOVARY[b] and WILSON SAVINO[c]

[a]*Department of Immunology, Basic Research Center, National Cancer Institute of Rio de Janeiro*; [b]*Department of Biochemistry, Institute of Biology, Rio de Janeiro State University*; [c]*Laboratory on Thymus Research, Oswaldo Cruz Foundation, Rio de Janeiro*

*(Received 22 July 1996; In final form 29 April 1997)*

In the present study, we used the fetal organ culture (FTOC) technique in order to study a putative effect of epidermal growth factor (EGF) on the thymus ontogeny. Functional EGF receptors and more recently the EGF molecule itself, respectively, on the membrane of epithelial components of thymic stroma and on a few thymocytes in adult thymus, had been reported in the literature. We could observe a dose-dependent decrease in cellularity and a progressive retention of thymocytes in the double-negative ($CD4^-/CD8^-$) stage of differentiation when exogenous EGF was added. Epidermal growth factor interfered with both fetal stroma growth and thymocyte development at a precise moment, that is, in the passage from double-negative to the double-positive ($CD4^+/CD8^+$) stage. After a 7-day FTOC in the presence of EGF, most cells recovered were Thy-$1.2^+$, c-$kit^+$, $TSA1^{-/int}$, $CD3^-$, and one of $CD44^{high}/CD25^{int}$, $CD44^-/CD25^{int}$, or $CD44^-/CD25^-$. Some developed into $\gamma\delta TCR^+$ cells with a mature ($CD3^+$) phenotype, but not into $\alpha\beta TCR^+$ thymocytes. It seems that EGF addition makes the cultures "nonpermissible" for $\alpha\beta TCR^+$ thymocyte generation. We report here the presence of a high Mr "EGF-like" molecule on the membrane of fetal thymocytes, which role in the observed effects is under investigation. Further biochemical characterization of this molecule is still required, because its presence was only evidenced on the basis of its antigenicity.

*Keywords:* Epidermal growth factor, fetal thymus organ culture, T-cell ontogeny

### INTRODUCTION

Thymus ontogeny is marked by a sequence of events that lead the precursors derived from fetal liver or bone marrow to a mature T-cell stage (Godfrey and Zlotnik, 1993; Anderson and Perlmutter, 1995). In the mouse, colonization starts at 10 to 11 days of fetal life (Palacios and Samaridis, 1991), being succeeded by growth and differentiation of both thymocyte precursors and stromal cells, whose specialization depends on a mutual signaling, in a symbiotic relationship (Ritter and Boyd, 1993; Holländer et al., 1995). This cross-talk is mediated by soluble cytokines and hormones, direct contact through membrane

*Corresponding author. Present address: Claudia S. Freitas, Instituto Nacional de Cancer, Pesquisa Básica/6° andar, Praça Cruz Vermelha 23, Rio de Janeiro 20230-130 RJ Brasil.

molecules, and extracellular matrix deposition (Boyd et al., 1993; Savino et al., 1993), and is still poorly understood at least for the initial, pre-T-cell receptor expression stages. Fetal thymus organ cultures (FTOCs) allow these stages to be investigated, because after 7 to 12 days *in vitro*, starting with 13- to 15-day thymuses, the colonized intact organs develop mimicking almost entirely the *in vivo* situation up to delivery (Ceredig, 1988), which occurs around 20 days of fetal life. We used this technique to investigate a possible role for epidermal growth factor (EGF) in the thymus, because functional EGF receptors had been demonstrated on the membrane of the epithelial components of thymic stroma (Le et al., 1991), and a few cells in the adult thymus were shown by immunohistochemistry to express the EGF molecule itself (Screpanti et al., 1995).

EGF and its receptor are ubiquitously distributed in mammalian tissues (Carpenter, 1993). Curiously, they had been reported to be missing in mature hematopoietic cell types (Carpenter, 1993), until the synthesis and exportation of a member of the family, heparin-binding EGF, was shown in human peripheral blood T lymphocytes (Blotnick et al., 1994). Soluble EGF is present in many body fluids and produced in large quantities by platelets and submaxillary glands in the adult male mouse, physiologically displaying a circadian variation in submaxillary glands, but not in plasma (Krieger et al., 1976), where its levels reach a concentration as high as 152 ng/mL after an $\alpha$-adrenergic stimulus (Byyni et al., 1974; Grau et al., 1994). The small peptide, with a Mr of approximately 6000, results from the proteolytic cleavage of a larger membrane complex (Mr 74,000), which consists of two EGF peptides bearing a carboxyl-terminal arginine residue linked to two molecules of EGF-binding protein, an arginin esteropeptidase (Carpenter and Cohen, 1979). Its precursor, prepro-EGF, has an Mr of approximately 130,000 (Gray et al., 1983), and in some tissues such as kidneys, it is not cleaved but expressed as a transmembrane integral protein (Rall et al., 1985), for which a putative receptor function has been suggested (Carpenter, 1993).

We describe here a blockade of thymocyte growth and differentiation on the addition of exogenous EGF to FTOC, and the presence of a high Mr "EGF-like" molecule on the membrane of fetal thymocytes, which persists in the adult thymus roughly up to the passage to the double-positive $CD4^+/CD8^+$ stage, suggesting that the EGF system plays a pivotal role in thymus ontogeny at least during these early stages of differentiation.

### RESULTS

### Effects of Exogenous EGF Addition to Fetal Thymuses *In Vitro*

When 14-day fetal thymuses were exposed to natural murine EGF, a dose-dependent decrease in cellularity and a retention of thymocytes in the double-negative (DN; $CD4^-/CD8^-$) stage of differentiation could be observed (Figure 1). Such effect depended on the maintenance of EGF throughout the 7 days of culture, because growth and differentiation resumed when EGF was subtracted on day 3 of culture, and stopped when EGF was added on this day to control cultures (Figure 2A). The most primitive cells colonizing fetal thymus, however, seemed to survive even to 7 days of culture in the presence of 100 ng/mL EGF, whose subtraction allowed thymocytes to resume their differentiation process (Figure 2B), although with a *lag* phase longer than 1 week. Yet, fully developed FTOC were not seen, with respect to percentages of single-positive subsets, even for longer periods in the absence of EGF.

As reported in the literature, fetal thymocytes can only grow *in vitro* when the relationship between them and the stromal-cell types is maintained in the intact organ architecture (Watson et al., 1989). Lobe submersion cultures (LSC), in which the organs are cultured and submerged in medium, result in thymocyte death unless IL-7 and/or IL-2 are added (Watson et al., 1989). In this case, both cytokines restore growth of the less differentiated thymocyte subpopulations, but cannot restore differentiation up to the double-positive (DP; $CD4^+/CD8^+$) stage (Watson et al., 1989). In our experiments, the growth of thymocytes in LSC in the presence of IL-2 plus IL-7 was unaffected by EGF (data not shown). The growth of epithelial stromal elements over the substrate, however, was profoundly impaired in the presence of EGF (Figure 3). In the case of the EGF-driven blockade of FTOC, even at a suboptimal dose (20 ng/mL), neither normal growth nor differentiation of thymocytes could be resumed by the addition of IL-2 plus IL-7, and again only the most immature subsets were expanded (Figure 4A).

It was shown that DN thymocytes bear minute quantities of membrane-bound CD3$\epsilon$, capable of switching intracellular signals, and treatment with anti-CD3$\epsilon$ antibodies can lead thymocytes from immunodeficient mice to advance in their way to differentiation (Levelt et al., 1993a, 1993b; Jacobs et al., 1994; Shinkai and Alt, 1994). We then tried to overcome the blocking effects of EGF by adding anti-CD3$\epsilon$ antibodies to FTOC. This procedure led part of the immature DN thymocytes one step further, that is, to the CD4$^{low}$/CD8$^{+}$ phenotype (Figure 4B), but not to the DP stage.

The progression of immature thymocytes to the DP stage was demonstrated to occur in short-term suspension cultures involving cell division, but independent of an intact stromal compartment, which in fact negatively regulates this step (Takahama et al., 1994). Fetal thymus lobes from 14-day-old fetuses were cultured intact (FTOC) for 24 h, either in the presence or absence of 100 ng/mL EGF. Suspensions

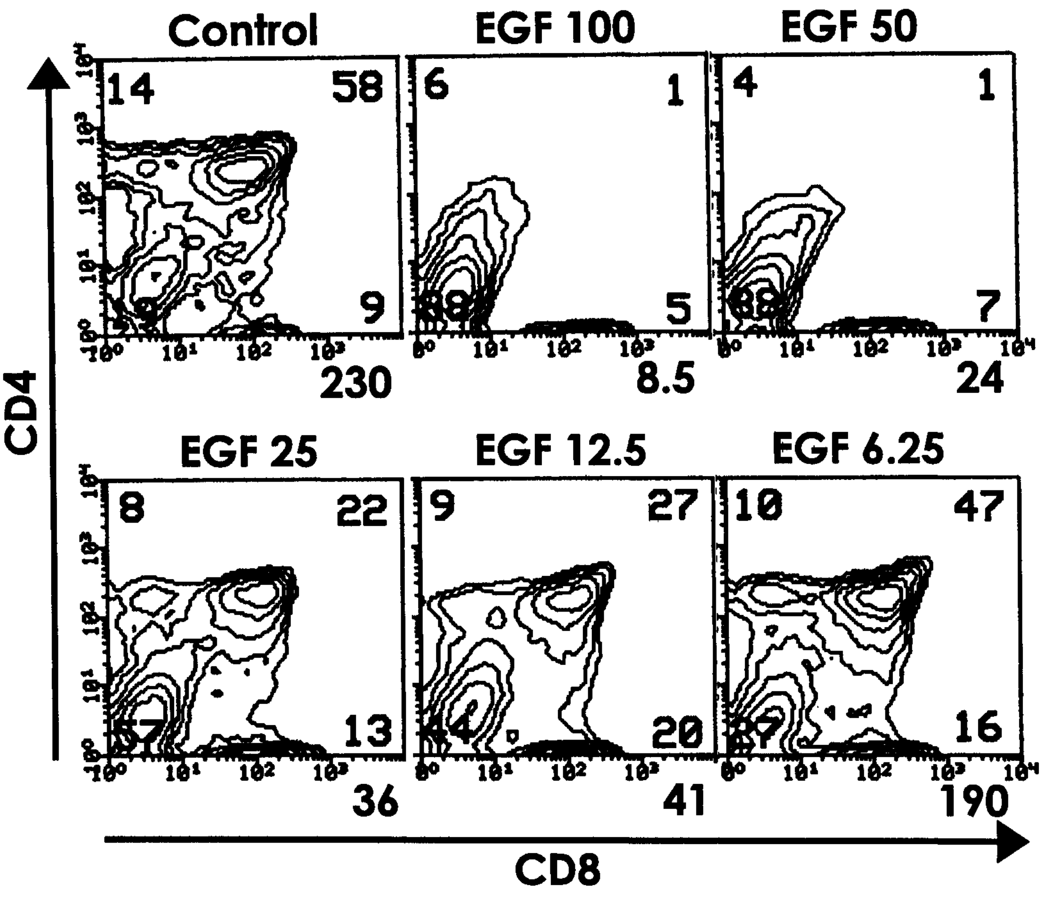

FIGURE 1 Dose-response of mnEGF on FTOC. Fourteen-day fetal thymic lobes were cultured intact as described in Materials and Methods. Exogenous mnEGF (ng/mL) was added at the beginning of culture and cells were harvested 7 days later for cytofluorometric analysis of CD4/CD8 expression. Values within each panel represent percentages, whereas mean cellularity per lobe is seen at the bottom right. Each point represents a pool of eight lobes. Similar results were observed in two further experiments.

were made from both pools of lobes, and 5 × $10^5$ cells from each separate pool were seeded in 24-well plates for 16 h in 1 mL complete medium, either in the presence or absence of 100 ng/mL EGF. After 16 h of culture, the four suspensions (C/C: control FTOC plus 16 h without EGF; C/EGF: control FTOC plus 16 h with EGF; EGF/C: EGF-FTOC plus 16 h without EGF; and EGF/EGF: EGF-FTOC plus 16 h with EGF) were double-stained with anti-CD4-PE and anti-CD8-FITC antibodies and differentiation assessed by cytofluorometric analysis. Results are described in Figure 5, which depicts one representative experiment. Recovery ranged from 62% in C/C to 46% in EGF/EGF cells. The profiles of CD4/CD8 staining were closely similar among the pairs, but whereas in the C/C and C/E suspension cultures, around 20% of the survivers showed a DP phenotype, in the EGF/C and EGF/EGF cultures, we could not observe a similar transition to the DP phenotype, suggesting that exogenous EGF acted in the first 24 h of culture, in the intact organ, instead of directly in the suspension cultures.

Taken together, these data argue against a general toxic effect of exogenous EGF on thymocytes,

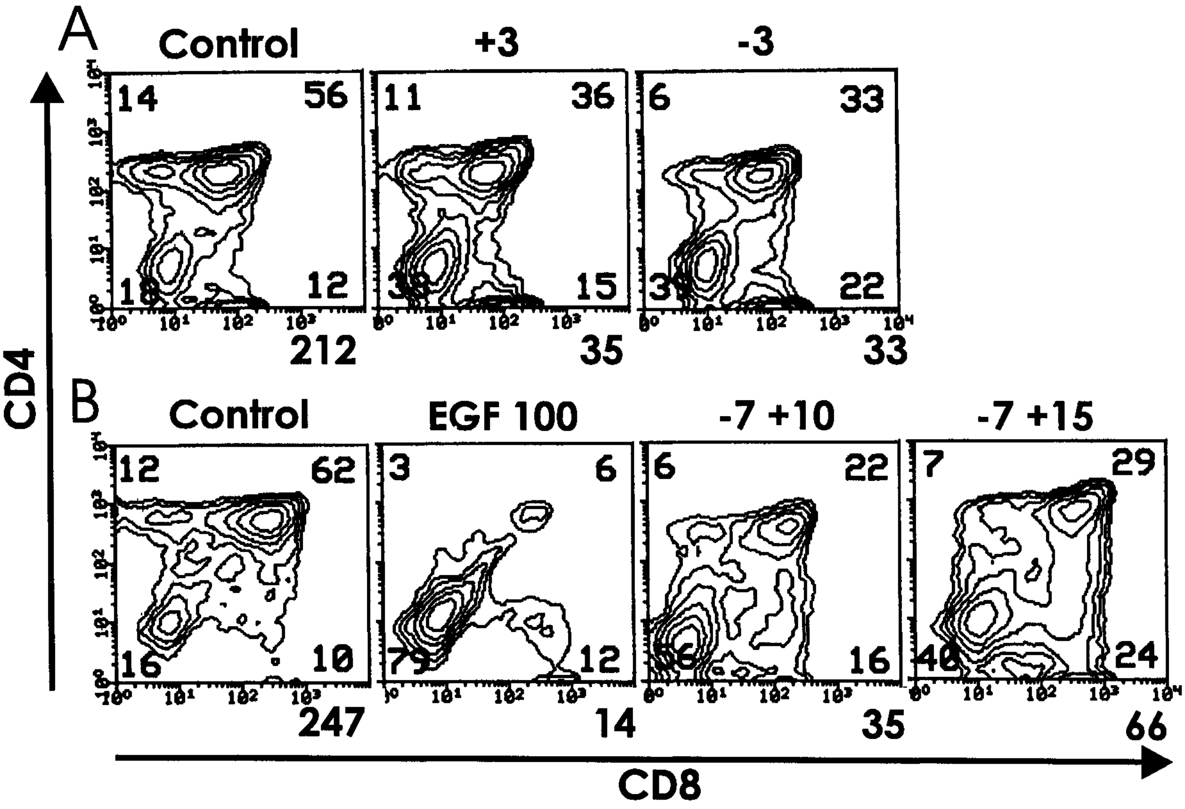

FIGURE 2 EGF effects on FTOC depends on its maintenance in culture media. (A) (Control): FTOC in the absence of EGF for 7 days. (+3): EGF (100 ng/mL) was added on the third day of culture, and cells were harvested on the seventh day. (−3): EGF was added at the beginning of culture and subtracted on the third day, the cells being harvested on the seventh day. Values within each panel represent percentages, and mean cellularity per lobe is seen at bottom right. Each point represents a pool of five lobes. Similar results were observed in two further experiments. (B) (Control): FTOC in the absence of EGF for 7 days; (EGF): FTOC in the presence of EGF for 7 days. (−7 + 10): EGF was added at the beginning of culture and subtracted at the seventh day, and the cultures were maintained for 10 additional days. (−7 + 15): EGF was added at the beginning of culture and subtracted at the seventh day, and the cultures were maintained for 15 additional days. Each point represents a pool of six lobes. Similar results were observed in two further experiments.

suggesting that EGF rather interferes with a precise checking point in thymocyte development, affecting both the stromal structure and the thymocyte subsets.

### Surface Characteristics of Thymocytes Recovered from a 7-day EGF-FTOC

The cells recovered from 7-day EGF-FTOC (100 ng/mL EGF) are Thy-1.2$^+$ (not shown), the majority being distributed among CD3$^-$/CD44$^{high}$/CD25$^{int}$, CD3$^-$/CD44$^-$/CD25$^{int}$, and CD3$^-$/CD44$^-$/CD25$^-$ (Figure 6). Most are c-kit$^+$ (as the most primitive cells colonizing the thymus) (Godfrey and Zlotnik, 1993) and EGF$^{int}$ (Figure 7A), TSA1$^{-/int}$ (a lymphostromal molecule that was shown to regulate early thymocyte development) (Randle et al., 1993) (Figure 7B), and some develop into $\gamma\delta$TCR$^+$ thymocytes with a mature phenotype (CD3$^+$, not shown). In fact, it seems that EGF addition makes the culture "nonpermissible" for $\alpha\beta$TCR$^+$ thymocyte generation (Figure 7C).

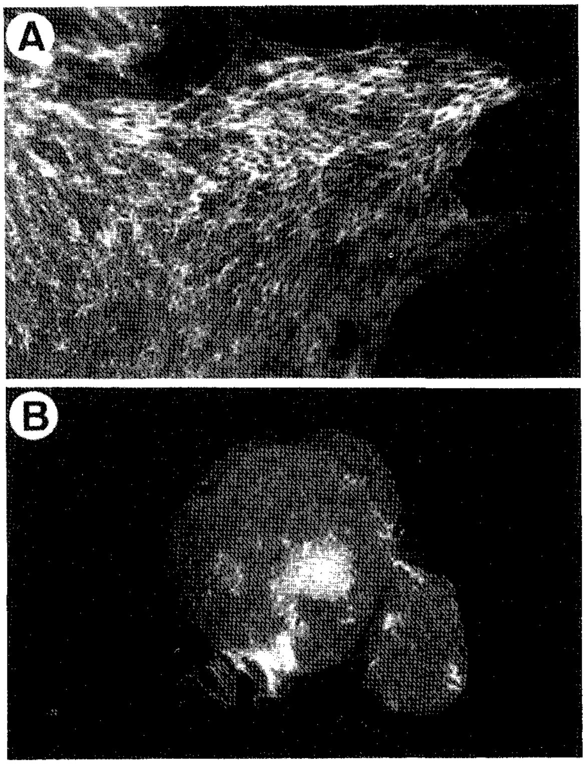

FIGURE 3 EGF blocks growth of epithelial cells in explanted fetal thymus lobes. Fourteen-day aged fetal thymus lobes (five to six per plate) were submerged in complete medium in the absence (**A**) or presence (**B**) of 100 ng/mL EGF for 20 days. Cultures were then fixed with methanol and immunostained with polyclonal rabbit anti-pan-cytokeratin antiserum followed by FITC-conjugated goat anti-rabbit IgG Abs (260 × magnification). Similar results were obtained in three further experiments.

### Recognition of EGF-like Molecules on the Surface of Immature Thymocytes

Cytofluorometric analysis of thymocyte suspensions from 14-day fetuses (mostly DN), incubated with rabbit polyclonal anti-EGF antiserum followed by FITC-conjugated anti-rabbit IgG Abs, revealed positive staining, which was also present in the DN subsets of young adult thymus and of control FTOC thymocytes (Figure 8A). Curiously, a less bright pattern of anti-EGF staining (logarithmic scale) was observed for the DN thymocytes recovered from a 7-day EGF-FTOC (100 ng/mL EGF), compared to fresh fetal thymocytes, and that did not seem to accompany the reduction in cell size (linear scale) (Figure 8B).

When fetal thymocyte suspensions from 14- and 16-day fetuses were submitted to SDS-PAGE, blotted and revealed with anti-EGF rabbit antiserum followed by PA conjugated anti-rabbit Abs, we could observe a band with an approximate Mr of 120,000 (Figure 9) which was not present in extracts from other fetal or adult tissues (heart and liver), and could be only observed in adult thymocyte extracts when as many as $10^7$ cells were extracted for analysis (not shown). When the same extracts were run under reducing conditions ($\beta$-mercaptoethanol in the sample buffer) and the blot developed with the same antiserum, the 120,000-Mr band was not observed, nor was it observed when the nonreducing electrophoresis was blotted and developed with normal rabbit serum as control (not shown).

The preceding data suggest that an EGF-like immunoreactive molecule is present on the surface of immature thymocytes and is possibly implicated in the effects observed following exogenous EGF addition.

### DISCUSSION

We report here a blockade of fetal thymocyte development *in vitro* on the addition of exogenous EGF to FTOC. The effect is dose-dependent and requires the constant presence of EGF, a fact that prevents the evaluation of the influence of this factor on each cellular compartment separately, in lobe reconstitution experiments using EGF-preincubated cells, for example. It was apparent, however, that both thymocyte and stromal compartments were affected, possibly at their interaction, because in LSC, the growth of the cytokeratin-positive cell subset was blocked on EGF addition, whereas the growth of immature thymocytes, which depends on IL-2 and/or IL-7 addition and is rather independent of stromal interaction (Watson et al., 1989), was not affected by EGF (not shown).

With respect to the thymocyte compartment, a blockade in differentiation seemed to occur in FTOC, because the tentative reversal of EGF effects by the addition of IL-2 and IL-7 to FTOC-EGF did in fact expand the immature subset, but could not reverse the retention imposed by this factor in the passage to the DP stage (Figure 4A). Figure 5 reinforces this conclusion, because this step was effectively blocked by the action of EGF in the intact organ but not in the thymocytes freed from stromal interactions, a fact arguing against a general toxic effect. Even anti-CD3$\epsilon$ MoAb, used to lead the DN phenotype into DP in

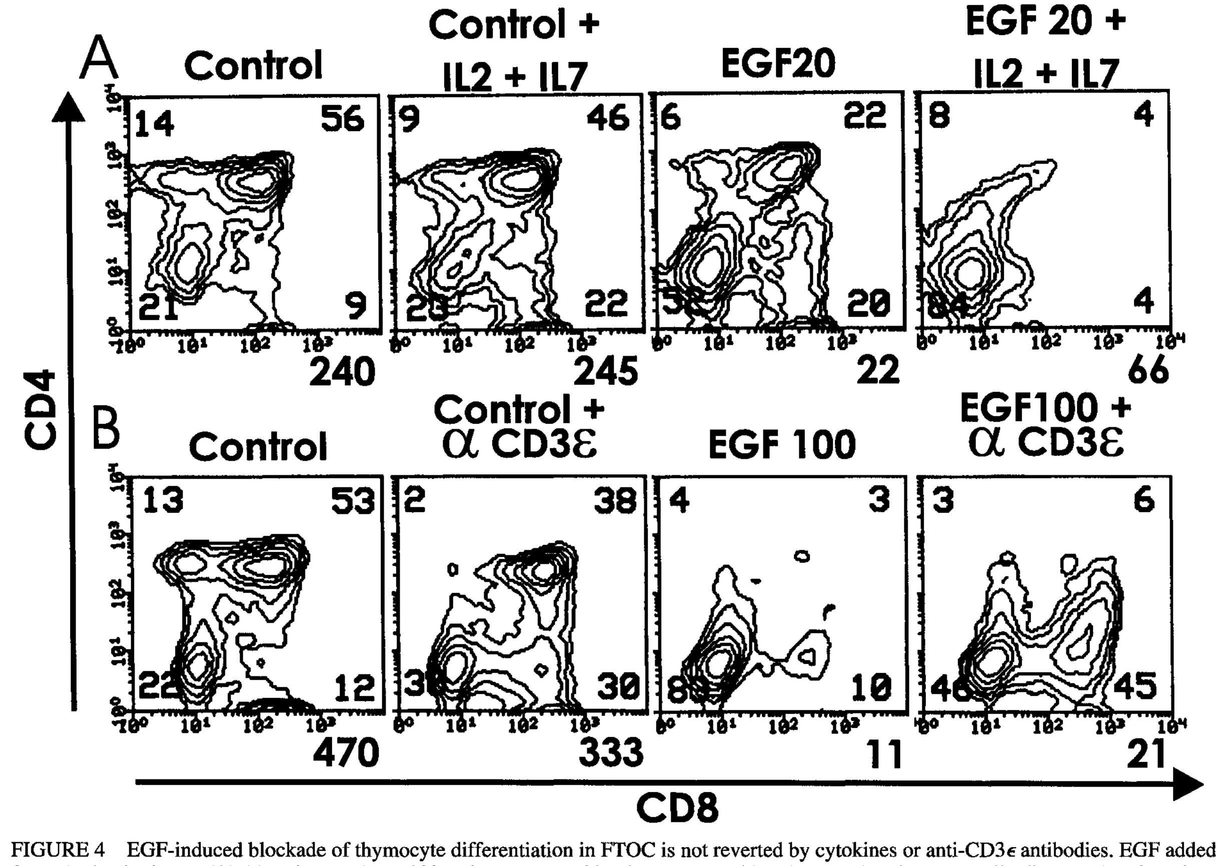

FIGURE 4 EGF-induced blockade of thymocyte differentiation in FTOC is not reverted by cytokines or anti-CD3$\epsilon$ antibodies. EGF added from the beginning at (**A**) 20 ng/mL and (**B**) 100 ng/mL; IL-2 at 20 U/mL, IL-7 at 20 ng/mL; and anti-CD3$\epsilon$ antibodies at 25 $\mu$g/mL final concentration. Values within each panel represent percentages, and mean cellularity per lobe is seen at bottom right. Each point represents a pool of (**A**) 5 and (**B**) 8 lobes. Similar results were observed in two further experiments.

immunodefficient mice (Levelt et al., 1993a, 1993b; Jacobs et al., 1994; Shinkai and Alt, 1994), pushed DN cells up to an intermediate stage, that is, $CD4^{low}$/$CD8^{+}$, but without overcoming the EGF-imposed blockade in the transition to the DP phenotype (Figure 4B).

When we analyzed the surface phenotype of the remaining FTOC-EGF immature cells compared to that of fetal thymocytes, we found that an EGF-like immunoreactive molecule was present in the latter but had its expression decreased in the former, whereas a similar decrease could not be observed with other surface molecules, such as c-kit (Figures 7 and 8). This raised the hypothesis that "membrane-EGF" may take part in the observed effects. The "EGF-bearing" cells would either be aborted, the exogenous EGF acting as an inhibitor of a necessary interaction of the membrane-immobilized molecule with EGF receptors in neighboring cells, or prevented from expressing this molecule (downregulation) due to the lack of this interaction. This putative relationship was reinforced because in adult young thymuses, the surface anti-EGF staining remains present in the DN thymocyte subset up to the transition to the DP stage (Figure 8). Within this context, knowing that epithelial components of young thymic stroma express EGF receptors (Le et al., 1991; Screpanti et al., 1995), one may assume that the "EGF system" takes part in the cross-talk between immature thymocytes and these stromal elements, not only during fetal life, but also in the physiology of the thymus after birth. Although the exogenous EGF-driven blockade of thymocyte differentiation described here worked as a tool for demonstrating a putative physiological event that still remains to be further clarified, it should be mentioned that the maximal EGF dose used (Figure 1) is still below the plasma EGF levels reached in mice subjected to an $\alpha$-adrenergic stimulus (Byyni et al., 1974; Grau et al., 1994), and therefore it remains possible that under this circumstance an *in vivo* blockade can also occur.

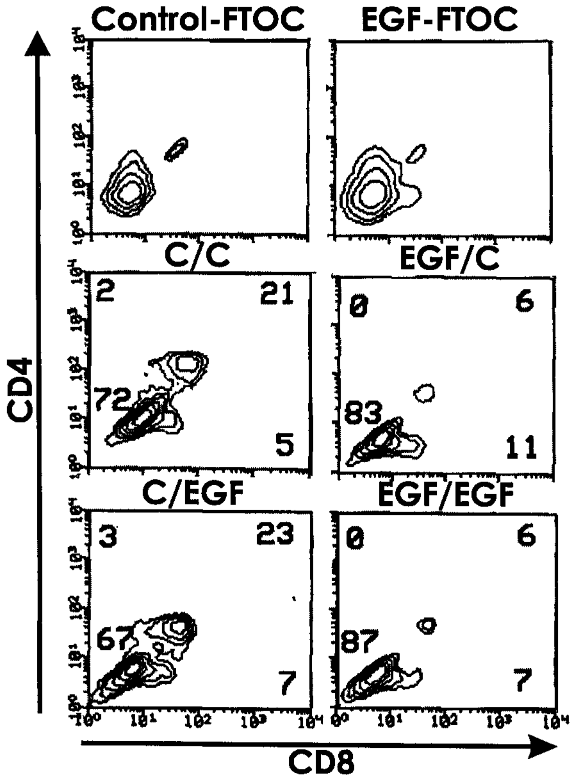

FIGURE 5 Cytofluorometric analysis of CD4/CD8 expression after a 16-h suspension culture of immature thymocytes. Fetal thymus lobes were first cultured intact in the absence (**Control-FTOC**) or presence (**EGF-FTOC**) of 100 ng/mL EGF for 24 h. The lobes were smashed, suspensions incubated for 1 h in complete medium in order to exclude adherent cells. Aliquots were immunostained for CD4/CD8, and the remaining cells were seeded ($5 \times 10^5$ in 1 mL of complete medium) in 24-well plates and incubated for 16 additional hours either in the absence or in the presence of 100 ng/mL EGF before being harvested and evaluated by flow cytometry. (**C/C**): 24-h Control-FTOC plus a 16-h suspension culture without EGF. (**C/EGF**): 24-h Control-FTOC plus a 16-h suspension culture containing EGF. (**EGF/C**): 24-h EGF-FTOC plus a 16-h suspension culture without EGF. (**EGF/EGF**): 24-h EGF-FTOC plus a 16-h suspension culture with EGF. Values within each panel represent percentages from total living cells. Similar results were observed in two further experiments.

Surprisingly, the addition of exogenous EGF to submerged fetal thymus cultures impaired the growth of epithelial (cytokeratin-positive) cells from the explanted organs over the substrate, instead of acting as a growth factor, as has been reported for soluble EGF added to thymic epithelial cells (Le et al., 1991). It seemed puzzling that a growth factor inhibited instead of enhancing the growth of its target cell, unless its presence was in fact impairing a preexisting signal that occurred physiologically. This apparently occurs for stem-cell factor (SCF; c-kit ligand) (Miyazawa et al., 1995). In this case, a transmembrane protein precursor generated a more persistent signal

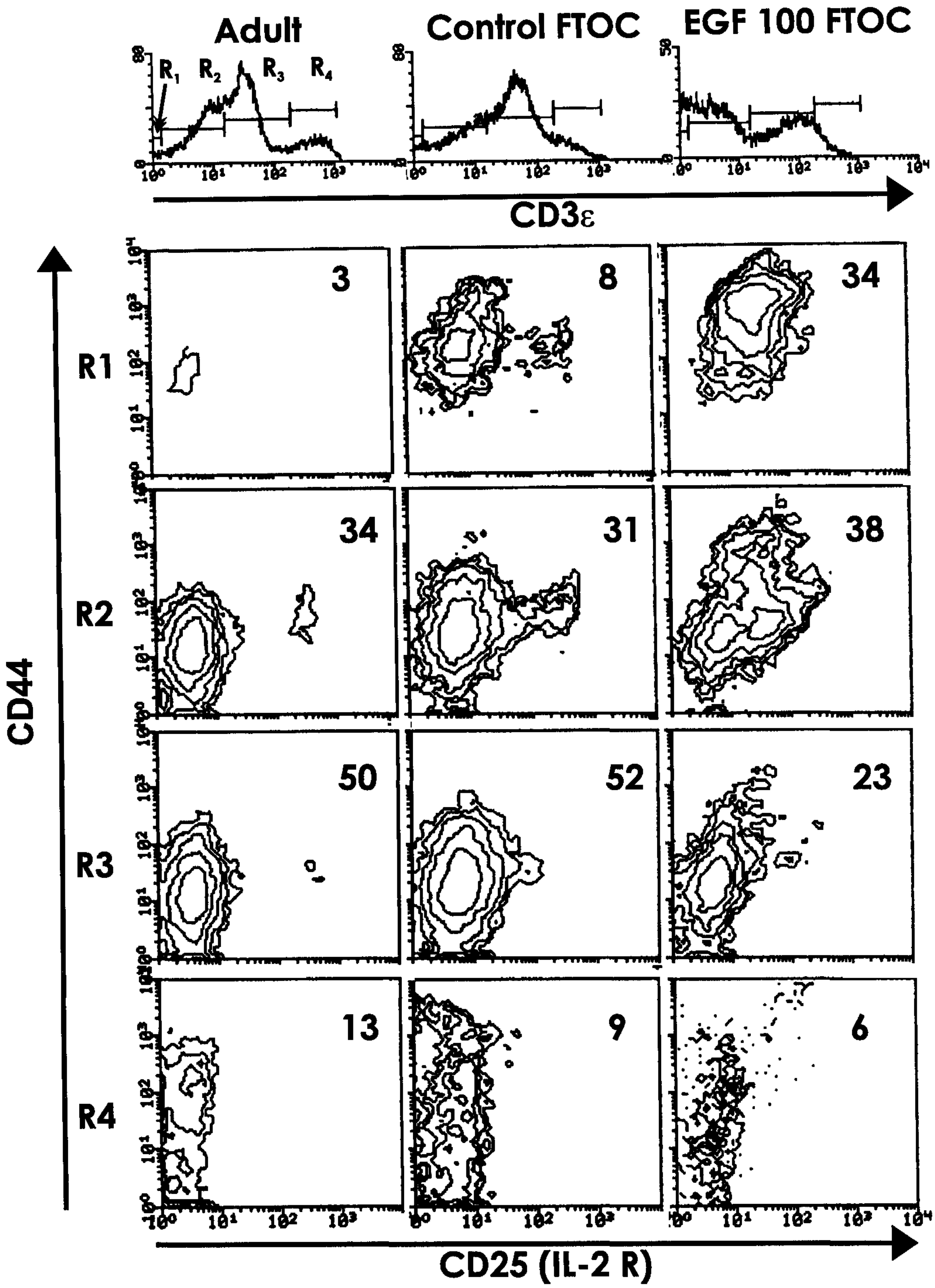

FIGURE 6 Surface characteristics of the thymocytes recovered after 7-day culture in the presence of 100 ng/mL EGF, compared to Control-FTOC thymocytes and young adult mouse thymocytes. Triple staining consisted of anti-CD25 (IL-2R)-FITC MoAb, anti-CD44-quantum red MoAb, and anti-CD3ε biotin MoAb+ PE-conjugated streptavidin. Plots in R1, R2, R3, and R4 represent analysis of CD44/CD25 patterns for regions from anti-CD3ε histograms, in which R1 represents the first region at left (negative). Values within each panel represent percentages from total living cells. Similar results were observed in two further experiments.

than the soluble one, which was transient due to prompt internalization with the receptor. Biologically active transmembrane precursors have also been demonstrated for macrophage colony-stimulating factor, IL-1$\alpha$ and TGF$\alpha$ (a member of the EGF family that shares with it the same receptor) (Massagué, 1983; Kurt-Jones et al., 1985; Beuscher et al., 1987; Brachman et al., 1989; Wong et al., 1989; Anklesaria et al., 1990; Stein et al., 1990; Teixidó et al., 1990). It seems possible that in the fetal thymic microenvironment, EGF acts similarly to SCF in bone marrow, so that soluble EGF may actually "switch

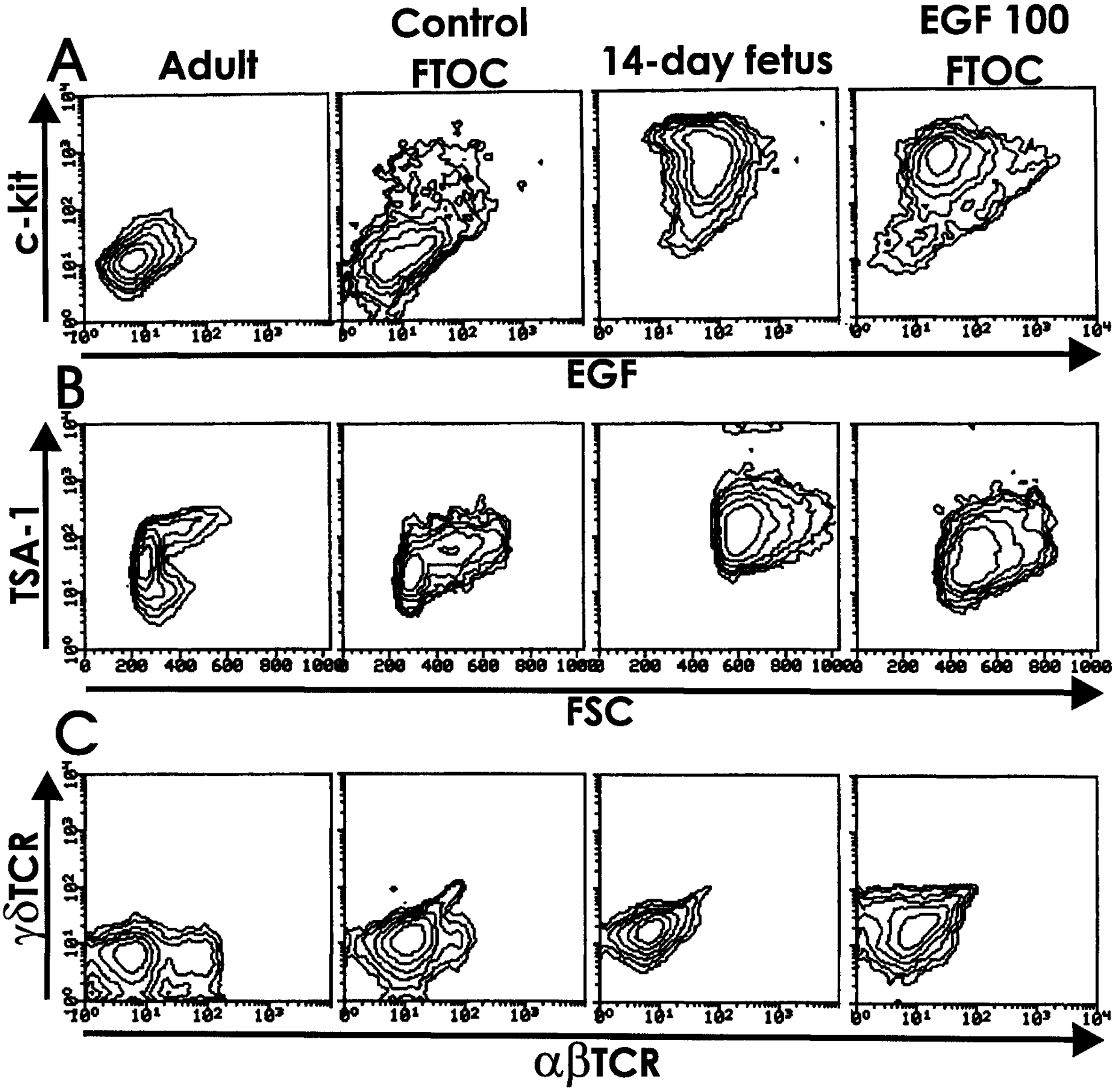

FIGURE 7 Surface characteristics of the thymocytes recovered after 7-day culture in the presence of 100 ng/mL EGF, compared to Control-FTOC thymocytes, 14-day fetal thymocytes, and young adult mouse thymocytes. Double-stainings consisted of anti-c-kit-PE MoAb × rabbit anti-EGF antiserum followed by FITC-conjugated goat anti-rabbit IgG Abs, and anti-$\alpha\beta$ TCR-FITC MoAb × anti-$\gamma\delta$ TCR-biotin MoAb followed by tricolor-conjugated streptavidin. Anti-TSA1-FITC MoAb single staining is plotted against size (FSC), and each point represents a pool of six, eight, and six lobes, respectively.

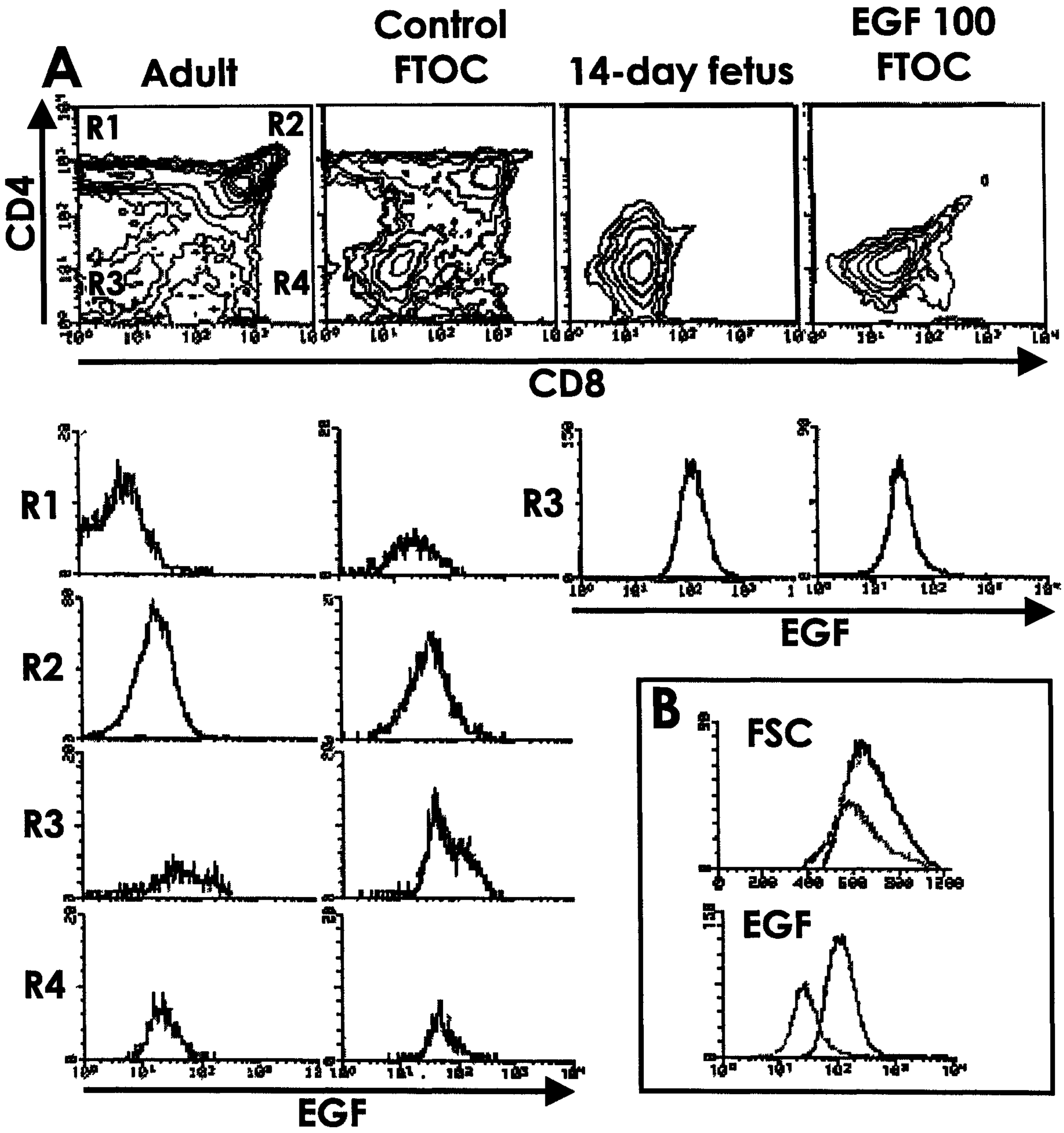

FIGURE 8 Cytofluorometric analysis of anti-CD4/CD8/EGF triple staining. Profiles from normal adult, 14-day fetal, Control-FTOC, and EGF-FTOC (100 ng/mL EGF) thymocytes are shown. (A) Histograms represent anti-EGF profiles for each thymocyte subset defined by CD4/CD8 analysis (contour plots). (B) Comparison of 14-day fetal thymocytes (black) and EGF-FTOC thymocytes (after a 7-day culture; gray) in terms of intensity of anti-EGF staining (log scale) and size (linear scale). Each point represents a pool of 12 lobes.

off" the physiological task of a membrane-immobilized "EGF-like" molecule.

It should be mentioned that exogenous TGF$\alpha$ (100 ng/mL; human recombinant; Gibco/BRL) did also affect FTOC in a manner similar to that seen with EGF (data not shown), as did tyrphostins (unpublished results), specific blockers of EGF-receptor tyrosine kinase activity (Levitzki and Gazit, 1995), which suggests the intermediation of EGF receptors in the mentioned blockade. Also, recombinant human EGF (> 98% pure by HPLC; Gibco/BRL) was as effective as, or even more effective than, natural murine EGF (data not shown), showing that what we observed was not caused by another substance present in trace amounts as a contaminant.

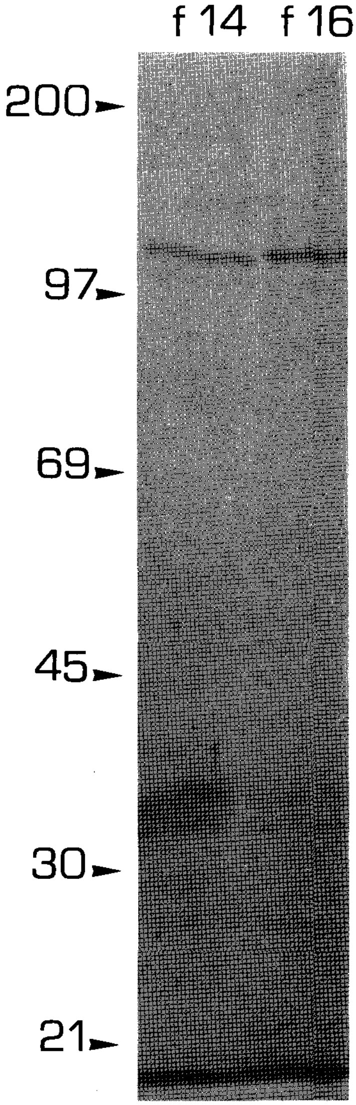

FIGURE 9 Presence of a high Mr, anti-EGF immunoreactive molecule in fetal thymocytes. Western blot of 14-day aged (F14) and 16-day aged (F16) fetal thymocytes. Extracts were made using $6 \times 10^5$ cells, which were solubilized and boiled in sample buffer under nonreducing conditions, run on a 7.5% SDS-PAGE, blotted onto nitrocellulose membranes, and developed with anti-EGF antiserum followed by PA-conjugated goat anti-rabbit IgG Abs. The position of the Mr standards ($10^{-3}$) is shown at left.

Within this context, it should be emphasized that although the epithelial growth was impaired in EGF-LSC, other cell types such as fibroblasts grew exuberantly over substrate (data not shown). One may assume that an imbalance among the thymic microenvironmental cell types was being imposed by EGF addition, favoring fibroblast growth. A role for mesenchimal cells besides epithelial ones in thymic cross-talk has been demonstrated (Anderson et al., 1993). However, the addition of another fibroblast growth-inducing activity, namely, basic fibroblast growth factor (Boehringer, Mannheim, Germany), did not produce effects similar to those of EGF on FTOC development (not shown). Because some $CD3^+$/$\gamma\delta TCR^+$ cells, but not $\alpha\beta TCR^+$ cells, developed in EGF-FTOC (Figure 7), it seems that this growth factor made the thymic microenvironment "non-permissible" for the development of $\alpha\beta TCR^+$ lineages. As suggested by Farr et al. (1990), the contribution of thymic environment to the differentiation of these distinct lineages seems to be qualitatively different.

Lastly, one should mention that immobilized EGF-like repeats are present in various extracellular matrix proteins (Engel, 1989; Yamada, 1991), including laminin and tenascin, which are constitutive components of the thymic microenvironment (Savino et al., 1993; Freitas et al., 1995) and have been shown to play important roles in cellular development in other systems (End et al., 1992; Schuppan and Rühl, 1994), besides modulating T-cell functions (Shimizu et al., 1990; Hemesath et al., 1994). Also adhesion molecules, such as P-selectin, contain immobilized EGF-like repeats, in this case responsible for leukocyte binding to endothelium (Gibson et al., 1995). Thus, for a better understanding of the role of the EGF system in thymus physiology, immobilized EGF-like repeats should be also evaluated.

### MATERIALS AND METHODS

### Animals

C57BL6/J mice from the animal facilities of the National Cancer Institute of Rio de Janeiro were used. Females were bred overnight, separated from males in the morning (day 0), and maintained on a diet supplemented with sunflower and corn seeds for 14 days. Pregnant females were killed by ether anesthesia, the uterus was excised, and fetuses were harvested under sterile conditions, and washed in HBSS (Sigma Co., St. Louis, MO).

### Fetal Thymus Organ Cultures (FTOC) and Lymphocyte Submersion Cultures

The fetal organ cultures were run as described in the literature (Ceredig, 1988) with some modifications. By using a watchmaker forceps, the thymuses were excised into Petri dishes containing culture medium (DMEM, with 2 g/L sodium bicarbonate, with osmolarity adjusted to 310 mOsm with NaCl) and further cleaned from residual contaminating tissues with the aid of a needle. Lobes were assembled (5 to 10 per dish) on a 0.22-$\mu$m Millipore membrane (previously boiled in a large volume of Milli-Q water) sustained by a stainless steel grid inside of a delta NUNC plate (Nunclon, Rosvilde, Denmark) containing 1.5 mL of culture medium with 10% FCS (defined serum, Hyclone, Logan, UT), glutamine, nonessential amino acids (Gibco/BRL; Life Technologies, Gaithersburg, MD), 60 mg/L penicillin and 100 mg/L streptomycin. Complete medium was changed every 3 days, and after 7 to 12 days of culture, lobes were harvested into a small volume of medium, smashed under a glass coverslip, and thymocytes were harvested and counted, being either suspended in ice-cold medium when processed for flow cytometry or washed in HBSS and dissolved in sample buffer when submitted to SDS-PAGE. For lymphocyte submersion cultures (LSC), lobes were explanted to the plates in the absence of the grid and membrane, being submerged in complete medium, and lymphocytes were harvested simply by flushing the medium at a given time, and the stromal adherent components were saline-washed and methanol-fixed for immunohistochemical analysis, as described in what follows. Murine natural EGF (mnEGF; from mouse submaxillary glands, a single band by Western blot using anti-mouse EGF; E-4127 Sigma) was used. Other cytokines used were human rIL-2, Hoffman-LaRoche (kindly given by Dr. Richard Peck, Basel Institute for Immunology), and murine rIL-7 (R&D Systems, Minneapolis, MN).

### Antibodies

Purified hamster anti-mouse CD3-$\epsilon$ MoAb (no azide/low endotoxin), clone 145-2C11; biotin-conjugated hamster anti-mouse CD3-$\epsilon$ MoAb; PE-conjugated rat anti-mouse CD117 (c-kit receptor) MoAb, clone 3C1; FITC-conjugated rat anti-mouse TSA1 MoAb, clone MTS35, and PE-conjugated hamster anti-mouse $\gamma\delta$TCR, clone GL3, were purchased from Pharmingen (San Diego). Rabbit polyclonal anti-mouse EGF antiserum; FITC-conjugated goat anti-rabbit IgG Ab; alkaline phosphatase (PA)-conjugated goat anti-rabbit IgG Ab; FITC-conjugated rat anti-mouse CD25 (IL-2R) MoAb, clone AMT13, and quantum red-conjugated rat anti-mouse CD44 (Pgp1) MoAb, clone IM7.8.1, were Sigma products. FITC-conjugated rat anti-mouse Thy 1.2 MoAb, clone 30-H12; PE-conjugated rat anti-mouse CD4 MoAb, clone GK1.5; FITC-conjugated rat anti-mouse CD8 MoAb, clone 53-6.7, and PE-conjugated streptavidin were obtained from Becton Dickinson (San Jose, CA). Tricolor-conjugated streptavidin and biotin-conjugated hamster anti-mouse TCR$\alpha\beta$ MoAb, clone H57-597, were purchased from Caltag (San Francisco) and Gibco/BRL, respectively.

### Flow Cytometry

Cells in ice-cold medium or HBSS plus 2% FCS were submitted to double or triple staining and then treated with propidium iodide at a final concentration of 2 $\mu$g/mL in order to exclude the dead cells. Acquisition was performed in a FACScan apparatus (Becton Dickinson) equipped with a 15-mW air-cooled 488-nm argon-ion laser. Fluorescein green fluorescence and PE or PI orange fluorescence were collected after 530- and 585-nm band-pass filters, respectively. Appropriate electronic compensation was applied between these fluorescence channels to remove spectral overlap. Data acquisition was carried out using the LYSYS II software program (BDIS) on a HP9000/300 Hewlett-Packard computer. Twenty thousand events were harvested from each sample.

### Electrophoresis and Western Blots

Fetal thymocytes, adult thymocytes or other tissues were dissolved and boiled in sample buffer and analyzed by 7.5% acrylamide-bisacrylamide SDS-PAGE, according to Laemmli and Favre (1973), under nonreducing conditions. Proteins were transferred in Towbin's buffer containing 0.1% SDS to 0.45-$\mu$m nitrocellulose membranes, which were blocked with 3% BSA in tris-buffered saline, and developed with rabbit polyclonal anti-mouse EGF antiserum, followed by PA-conjugated goat anti-rabbit IgG Abs, and with the Sigma Fast BCIP/NBT buffered substrate system (0.15 mg/mL 5-bromo-4-chloro-3-indolyl phosphate; 0.30 mg/mL nitro blue tetrazolium; 100 mM Tris; 5 mM $MgCl_2$). Mr standards were Rainbow Coloured Proteins (Amersham, Buckinghamshire, UK).

### Immunocytochemistry

Fourteen-day fetal thymus lobes were explanted and cultured for 20 days submerged in complete medium (half volume carefully changed every 3 days) inside 24-well culture plates. The plates were saline-washed, methanol-fixed, and stained with rabbit anti-pan-cytokeratin antiserum (Dako, Glostrup, Denmark), followed by FITC-conjugated goat anti-rabbit IgG Abs.

### *Acknowledgements*

The authors thank Mr. Claudio V. da Silva and Mr. Inaldo D. de Oliveira for the photographs, Dr. Joseli Lannes Vieira for some critical comments, and Dr. Ramza Cabral Harab for the FACS FIGs.

### *References*

Anderson G., Jenkinson E. J., Moore N. C. and Owen J. T. (1993) MHC class II-positive epithelium and mesenchime cells are both required for T-cell development in the thymus. *Nature*, **362**, 70-73.

Anderson S. J. and Perlmutter R. M. (1995) A signaling pathway governing early thymocyte maturation. *Immunol. Today*, **16**, 99-105.

Anklesaria P., Teixidó J., Laiho M., Pierce J. H., Greenberger J. S. and Massagué J. (1990) Cell-cell adhesion mediated by binding of membrane-anchored transformed growth factor $\alpha$ to epidermal growth factor receptors promotes cell proliferation. *Proc. Natl. Acad. Sci. USA*, **87**, 3289-3293.

Beuscher H. U., Fallon R. J. and Colten H. R. (1987) Macrophage membrane interleukin 1 regulates the expression of acute phase proteins in human hepatoma Hep 3B cells. *J. Immunol.*, **139**, 1896-1901.

Blotnick S., Peoples G. E., Freeman M. R., Eberlein T. J. and Klagsbrun M. (1994) T lymphocytes synthesize and export heparin-binding epidermal growth factor-like growth factor and basic fibroblast growth factor, mitogens for vascular cells and fibroblasts: Differential production and release by $CD4^+$ and $CD8^+$ T cells. *Proc. Natl. Acad. Sci. USA*, **91**, 2890-2894.

Boyd R. L., Tucek C. L., Godfrey D. I., Izon D. J., Wilson T. J., Davidson N. J., Bean A. G. D., Ladyman H. M., Ritter M. A. and Hugo P. (1993) The thymic microenvironment. *Immunol. Today*, **14**, 445-459.

Brachmann R., Lindquist P. B., Nagashima M., Kohr W., Lipari T., Napier M. and Derynck R. (1989) Transmembrane TGF-$\alpha$ precursors activate EGF/TGF-$\alpha$ receptors. *Cell*, **56**, 691-700.

Byyni R. L., Orth D. N., Cohen S. and Doyne E. S. (1974) Epidermal growth factor: Effects of androgens and adrenergic agents. *Endocrinology*, **95**, 776-782.

Carpenter G. (1993) EGF: New tricks for an old growth factor. *Cur. Opinion Cell Biol.*, **5**, 261-264.

Carpenter G. and Cohen S. (1979) Epidermal growth factor. *Ann. Rev. Biochem.*, **48**, 193-216.

Ceredig R. (1988) Differentiation potential of 14-day fetal mouse thymocytes in organ culture. Analysis of CD4/CD8-defined single-positive and double-negative cells. *J. Immunol.*, **141**, 355-362.

End P., Panayotou G., Entlwistle A., Waterfield M. D. and Chiquet M. (1992) Tenascin: A modulator of cell growth. *Eur. J. Biochem.*, **209**, 1041-1051.

Engel J. (1989) EGF-like domains in extracellular matrix proteins: Localized signals for growth and differentiation? *FEBS Lett.*, **251**, 1-7.

Farr A., Hosier S., Nelson A., Itohara S. and Tonegawa S. (1990) Distribution of thymocytes expressing $\gamma\delta$ receptors in the murine thymus during development. *J. Immunol.*, **144**, 492-498.

Freitas C. S., O'Campo Lyra J. S. P., Dalmau S. R. and Savino W. (1995) *In vivo* and *in vitro* expression of tenascin by human thymic microenvironmental cells. *Dev. Immunol.*, **4**, 139-147.

Gibson R. M., Kansas G. S., Tedder T. F., Furie B. and Furie B. C. (1995) Lectin and epidermal growth factor domains of P-selectin at physiologic density are the recognition unit for leukocyte binding. *Blood*, **85**, 151-158.

Godfrey D. I. and Zlotnik A. (1993) Control points in early T-cell development. *Immunol. Today*, **14**, 547-553.

Grau M., Rodriguez C., Soley M. and Ramirez I. (1994) Relationship between epidermal growth factor in mouse submandibular glands, plasma, and bile: Effects of catecholamines and fasting. *Endocrinology*, **135**, 1854-1862.

Gray A., Dull T. J. and Ullrich A. (1983) Nucleotide sequence of epidermal growth factor cDNA predicts a 128,000 molecular weight protein precursor. *Nature*, **303**, 722-725.

Hemesath T. J., Marton L. S. and Stefansson K. (1994) Inhibition of T cell activation by the extracellular matrix protein tenascin. *J. Immunol.*, **152**, 5199-5207.

Holländer G. A., Wang B., Nichogiannopoulou A., Platenburg P. P., vanEwijk W., Burakoff S. J., Gutierrez-Ramos J.-C. and Terhorst C. (1995) Developmental control point in induction of thymic cortex regulated by a subpopulation of prothymocytes. *Nature*, **373**, 350-353.

Jacobs H., Vandeputte D., Tolkamp L., deVries E., Borst J. and Berns A. (1994) CD3 components at the surface of pro-T cells can mediate pre-T cell development *in vivo*. *Eur. J. Immunol.*, **24**, 934-939.

Krieger D. T., Hauser H., Liotta A. and Zelenetz A. (1976) Circadian periodicity of epidermal growth factor and its abolition by superior cervical ganglionectomy. *Endocrinology*, **99**, 1589-1596.

Kurt-Jones E. A., Beller D. I., Mizel S. B. and Unanue E. R. (1985) Identification of a membrane-associated interleukin 1 in macrophages. *Proc. Natl. Acad. Sci. USA*, **82**, 1204-1208.

Laemmli U. K. and Favre M. (1973) Maturation of the head of bacteriophage T4. I. DNA packaging events. *Mol. Biol.*, **80**, 575-599.

Le P. T., Lazorick S., Whichard L. P., Haynes B. F. and Singer K. H. (1991) Regulation of cytokine production in the human thymus: Epidermal growth factor and transforming growth factor a regulate mRNA levels of interleukin $1\alpha$ (IL-$1\alpha$), IL-$1\beta$, and IL-6 in human thymic epithelial cells at a post-transcriptional level. *J. Exp. Med.*, **174**, 1147-1157.

Levelt C. N., Ehrfeld A. and Eichmann K. (1993a) Regulation of thymocyte development through CD3. I. Timepoint of ligation of CD3$\epsilon$ determines clonal deletion or induction of developmental program. *J. Exp. Med.*, **177**, 707-716.

Levelt C. N., Mombaerts P., Iglesias A., Tonegawa S. and Eichmann K. (1993b) Restoration of early thymocyte differentiation in T-cell receptor $\beta$-chain-deficient mutant mice by transmembrane signaling through CD3$\epsilon$. *Proc. Natl. Acad. Sci. USA*, **90**, 11401-11405.

Levitzki A. and Gazit A. (1995) Tyrosine kinase inhibition: An approach to drug development. *Science*, **267**, 1782-1788.

Massagué J. (1983) Epidermal growth factor-like transforming growth factor. *J. Biol. Chem.*, **258**, 13614-13620.

Miyazawa K., Williams D. A., Gotoh A., Nishimaki J., Broxmeyer H. E. and Toyama K. (1995) Membrane-bound steel factor induces more persistent tyrosine kinase activation and longer life span of *c-kit* gene-encoded protein than its soluble form. *Blood*, **85**, 641-649.

Palacios R. and Samaridis J. (1991) Thymus colonization in the developing mouse embryo. *Eur. J. Immunol.*, **21**, 109-113.

Rall L. B., Scott J. and Bell G. I. (1985) Mouse prepro-epidermal growth factor synthesis by the kidney and other tissues. *Nature*, **313**, 228-233.

Randle E. S., Waanders G. A., Masciantonio M., Godfrey D. I. and Boyd R. L. (1993) A lymphostromal molecule, thymic shared Ag-1, regulates early thymocyte development in fetal thymus organ culture. *J. Immunol.*, **151**, 6027-6035.

Ritter M. A. and Boyd R. L. (1993) Development in the thymus: It takes two to tango. *Immunol. Today*, **14**, 462-469.

Savino W., Villa-Verde D. M. S. and Lannes-Vieira J. (1993) Extracellular matrix proteins in intrathymic T-cell migration and differentiation? *Immunol. Today*, **14**, 158-161.

Schuppan D. and Rühl M. (1994) Matrix in signal transduction and growth factor modulation. *Brazilian J. Med. Biol. Res.*, **27**, 2125-2141.

Screpanti I., Scarpa S., Meco D., Bellavia D., Stuppia L., Frati L., Modesti A. and Gulino A. (1995) Epidermal growth factor promotes a neural phenotype in thymic epithelial cells and enhances neuropoietic cytokine expression. *J. Cell. Biol.*, **130**, 183-192.

Shimizu Y., Van Seventer G. A., Horgan K. J. and Shaw S. (1990) Costimulation of proliferative responses of resting $CD4^+$ T cells by the interaction of VLA-4 and VLA-5 with fibronectin or VLA-6 with laminin. *J. Immunol.*, **145**, 59-67.

Shinkai Y. and Alt F. W. (1994) CD3$\epsilon$-mediated signals rescue the development of $CD4^+$ $CD8^+$ thymocytes in RAG-$2^{-/-}$ mice in the absence of TCR$\beta$ chain expression. *Int. Immunol.*, **6**, 995-1001.

Stein J., Borzillo G. V. and Rettenmier C. W. (1990) Direct stimulation of cells expressing receptors for macrophage colony-stimulating factor (CSF-1) by a plasma membrane-bound precursor of human CSF-1. *Blood*, **76**, 1308-1314.

Takahama Y., Letterio J. J., Suzuki H., Farr A. G. and Singer A. (1994) Early progression of thymocytes along the CD4/CD8 developmental pathway is regulated by a subset of thymic epithelial cells expressing transforming growth factor $\beta$. *J. Exp. Med.*, **179**, 1495-1506.

Teixidó J., Wong S. T., Lee D. C. and Massagué J. (1990) Generation of transforming growth factor-$\alpha$ from the cell surface by an O-glycosylation-independent multistep process. *J. Biol. Chem.*, **265**, 6410-6415.

Watson J. D., Morrissey P. J., Namen A. E., Conlon P. J. and Widmer M. B. (1989) Effect of IL-7 on the growth of fetal thymocytes in culture. *J. Immunol.*, **143**, 1215-1222.

Wong S. T., Winchel L. F., McCune B. K., Earp H. S., Teixidó J., Massagué J., Herman B. and Lee D. C. (1989) The TGF-$\alpha$ precursor expressed on the cell surface binds to the EGF receptor on adjacent cells, leading to signal transduction. *Cell*, **56**, 495-506.

Yamada K. M. (1991) Fibronectin and other cell interactive glycoproteins. In Cell Biology of Extracellular Matrix, Hay, E. D., Ed. (New York: Plenum Press), pp. 111-139.

